# Supplementary material for: From sequence to enzyme mechanism using multi-label machine learning
Source: BMC Bioinformatics. 2014 May 19;15:150. doi: 10.1186/1471-2105-15-150 (PMC4229970; doi:10.1186/1471-2105-15-150)
Supplement: Additional file 2 — Java code of ml2db. Additional file ml2db_code.tar.gz contains the Java source code to run the multi-label machine learning experiments and save the results to database. The code’s Javadoc is included. [file 1471-2105-15-150-S2.zip › additional file 2/ml2db/ml2db/doc/main/ml2db/mulan/mulanarff/IdMultiLabelInstances.html]

IdMultiLabelInstances


JavaScript is disabled on your browser.


- Overview
- Package
- Class
- Use
- Tree
- Deprecated
- Index
- Help

- Prev Class
- Next Class

- Frames
- No Frames

- All Classes

- Summary:
- Nested |
- Field |
- Constr |
- Method

- Detail:
- Field |
- Constr |
- Method


main.ml2db.mulan.mulanarff

## Class IdMultiLabelInstances

- java.lang.Object
- - mulan.data.MultiLabelInstances
  - - main.ml2db.mulan.mulanarff.IdMultiLabelInstances

- All Implemented Interfaces:
  :   java.io.Serializable

  ---

    

  ```
  public class IdMultiLabelInstances
  extends mulan.data.MultiLabelInstances
  implements java.io.Serializable
  ```

  A set of identified Mulan Multi Label instances (each instance has a unique
  string identifier). Known problem: make sure all class attributes come
  alphabetically before the feature attributes (append an uppercase 'A' to the
  class attribute names, if necessary)

  Version:
  :   11 Jul 2011

  Author:
  :   Luna De Ferrari ldf(at)st-andrews.ac.uk

  See Also:
  :   Serialized Form

- - ### Field Summary

    Fields

    | Modifier and Type | Field and Description |
    | --- | --- |
    | `static java.lang.String` | `EMPTY_INSTANCE_ATTRIBUTES_STRING` |
    | `static java.lang.String` | `EMPTY_STRING` |
  - ### Constructor Summary

    Constructors

    Constructor and Description

    `IdMultiLabelInstances(int arffId,
    DbManagers dbMan)`
  - ### Method Summary

    Methods

    | Modifier and Type | Method and Description |
    | --- | --- |
    | `void` | `addAll(IdMultiLabelInstances instances)` Instances must come from already compatible datasets! |
    | `boolean` | `addInstance(IdInstance instance)` |
    | `java.lang.String` | `addToInstanceNamesToAttributesStringMap(java.lang.String instanceName)` |
    | `java.lang.String` | `addToInstanceNamesToSparseAttrStringMap(java.lang.String instanceName)` |
    | `boolean` | `deleteInstance(java.lang.String name)` Remove an instance |
    | `java.lang.String` | `generateAttributesNamesString(weka.core.Instance i0, java.util.Set<weka.core.Attribute> attributes)` Generates a -- separated string with the name of all the non-zero valued attributes of an instance. |
    | `java.util.TreeSet<java.lang.String>` | `generateNonZeroFeatureAttributesOfTheseInstances()` Get the non-zero valued feature attributes of these instances |
    | `boolean[]` | `generatePartition(IdInstance instance, int[] labelIndices, int numberOfLabels)` The boolean[] partition of the labels for this instance (true if the label is present, false if absent, for each of the existing labels). |
    | `IdInstance` | `get(int index)` For test purposes only! slow! |
    | `weka.core.Instance` | `get(java.lang.String name)` |
    | `java.util.Vector<java.lang.String>` | `getAllAttributeNames()` |
    | `java.util.Vector<weka.core.Attribute>` | `getAllAttributes()` |
    | `java.lang.String` | `getArffFilePath()` |
    | `int` | `getArffId()` |
    | `java.util.ArrayList<weka.core.Attribute>` | `getAttributesArray()` |
    | `java.lang.String` | `getAttributesIndexString(IdInstance instance)` |
    | `java.lang.String` | `getAttributesNamesString(IdInstance instance)` |
    | `uk.ac.standrews.utils.main.stringutils.StringPool` | `getAttributesStringPool()` |
    | `weka.core.Instances` | `getByAttributesString(java.lang.String instanceAttributesString)` Finds an instance having all these attributes names as present (=1) attributes. |
    | `weka.core.Instances` | `getBySparseInstanceString(java.lang.String instanceString)` Find instances having the same sparse instance string ( indexed attributes string {0 1, 3 1, 5 1} including all feature and class attributes). |
    | `java.util.Set<weka.core.Attribute>` | `getClassAttributes()` |
    | `DbManagers` | `getDbMan()` |
    | `weka.core.Instance` | `getFirstInstance()` |
    | `java.util.TreeMap<java.lang.String,IdInstance>` | `getId2IdInstanceMap()` |
    | `java.util.TreeMap<java.lang.String,weka.core.Instance>` | `getId2WekaInstanceMap()` |
    | `java.util.Set<IdInstance>` | `getIdInstance(weka.core.Instance wekaInstance)` |
    | `IdInstance` | `getIdInstance(java.lang.String identifier)` |
    | `java.util.Vector<IdInstance>` | `getIdInstancesByAttributesString(java.lang.String instanceAttributesString)` |
    | `int` | `getIndexOfIdInstance(java.lang.String instanceName)` |
    | `java.util.TreeMap<IdInstance,boolean[]>` | `getInst2LabelsPartitionMap()` |
    | `static java.lang.String` | `getInst2PartitionMapString(java.util.TreeMap<IdInstance,boolean[]> inst2PartitionMap)` |
    | `uk.ac.standrews.utils.main.stringutils.StringPool` | `getInstanceNamesStringPool()` |
    | `uk.ac.standrews.utils.main.stringutils.StringPool` | `getInstanceStringPool()` |
    | `java.util.TreeMap<java.lang.String,java.lang.String>` | `getInstNames2AttrIndexStringMap()` |
    | `java.util.TreeMap<java.lang.String,java.lang.String>` | `getInstNames2AttrNamesStringMap()` |
    | `java.util.TreeMap<java.lang.String,java.lang.String>` | `getInstSparseStringToLabelsString()` |
    | `java.util.TreeMap<java.lang.String,boolean[]>` | `getLabels2PartitionMap()` |
    | `java.lang.String` | `getLabelsString(IdInstance inst)` Get a separated list of the labels assigned to this instance. |
    | `java.io.File` | `getLogFile()` |
    | `static java.util.TreeSet<java.lang.Integer>` | `getNonZeroAttribIndexesOfInstance(weka.core.Instance i0, java.util.Set<weka.core.Attribute> attributes)` Only input non-class (feature) attributes if the feature attributes indexes are desired. |
    | `static java.util.TreeSet<java.lang.String>` | `getNonZeroAttributeNamesOfAnInstance(weka.core.Instance i0, java.util.Set<weka.core.Attribute> attributes)` Get the non-zero valued attributes of an instance |
    | `java.util.TreeSet<java.lang.String>` | `getNonZeroLabelsOfAnInstance(weka.core.Instance i0)` Get the names of the non-zero valued class labels of an instance |
    | `java.util.TreeSet<java.lang.String>` | `getNonZeroLabelsOfTheseInstances()` Get the names of the non-zero valued class labels of these instances |
    | `java.util.Set<weka.core.Attribute>` | `getStoredFeatureAttributes()` |
    | `java.util.Set<IdInstance>` | `getToBePredicted()` |
    | `boolean[]` | `getTrueLabels(IdInstance idInstance)` |
    | `java.util.Vector<IdInstance>` | `getVector()` |
    | `uk.ac.standrews.utils.main.maputils.OneToManyMap<java.lang.String,IdInstance>` | `getWekaInst2IdInstMap()` |
    | `java.lang.String` | `getXmlFilePath()` |
    | `java.lang.String[]` | `labelNames()` |
    | `void` | `setClassAttributes()` |
    | `java.lang.String` | `toString()` |

    - ### Methods inherited from class mulan.data.MultiLabelInstances

      `clone, getCardinality, getDataSet, getDepth, getFeatureAttributes, getFeatureIndices, getLabelAttributes, getLabelDepth, getLabelDepthIndices, getLabelIndices, getLabelsMetaData, getLabelsOrder, getNumInstances, getNumLabels, hasMissingLabels, reintegrateModifiedDataSet`
    - ### Methods inherited from class java.lang.Object

      `equals, getClass, hashCode, notify, notifyAll, wait, wait, wait`

- - ### Field Detail


    - #### EMPTY\_INSTANCE\_ATTRIBUTES\_STRING

      ```
      public static java.lang.String EMPTY_INSTANCE_ATTRIBUTES_STRING
      ```


    - #### EMPTY\_STRING

      ```
      public static java.lang.String EMPTY_STRING
      ```
  - ### Constructor Detail


    - #### IdMultiLabelInstances

      ```
      public IdMultiLabelInstances(int arffId,
                           DbManagers dbMan)
                            throws mulan.data.InvalidDataFormatException
      ```

      Throws:
      :   `mulan.data.InvalidDataFormatException`
  - ### Method Detail


    - #### addAll

      ```
      public void addAll(IdMultiLabelInstances instances)
      ```

      Instances must come from already compatible datasets!

      Parameters:
      :   `instances` -


    - #### addInstance

      ```
      public boolean addInstance(IdInstance instance)
      ```


    - #### addToInstanceNamesToAttributesStringMap

      ```
      public java.lang.String addToInstanceNamesToAttributesStringMap(java.lang.String instanceName)
      ```


    - #### addToInstanceNamesToSparseAttrStringMap

      ```
      public java.lang.String addToInstanceNamesToSparseAttrStringMap(java.lang.String instanceName)
      ```


    - #### deleteInstance

      ```
      public boolean deleteInstance(java.lang.String name)
      ```

      Remove an instance

      Parameters:
      :   `name` - the unique instance identifier

      Returns:
      :   true if the instance could be deleted, false otherwise (because
          of error or instance not existing)


    - #### generateAttributesNamesString

      ```
      public java.lang.String generateAttributesNamesString(weka.core.Instance i0,
                                                   java.util.Set<weka.core.Attribute> attributes)
      ```

      Generates a -- separated string with the name of all the non-zero valued
      attributes of an instance. Eg. {IPR001--IPR002}

      Parameters:
      :   `i0` - an instance
      :   `attribute` - a set of attributes

      Returns:
      :   a -- separated string with the name of all the non-zero valued
          attributes of an instance. Eg. {IPR001--IPR002}


    - #### generateNonZeroFeatureAttributesOfTheseInstances

      ```
      public java.util.TreeSet<java.lang.String> generateNonZeroFeatureAttributesOfTheseInstances()
      ```

      Get the non-zero valued feature attributes of these instances

      Returns:
      :   the non-zero valued feature attributes of these instances


    - #### generatePartition

      ```
      public boolean[] generatePartition(IdInstance instance,
                                int[] labelIndices,
                                int numberOfLabels)
      ```

      The boolean[] partition of the labels for this instance (true if the
      label is present, false if absent, for each of the existing labels).

      Parameters:
      :   `instance` -

      Returns:
      :   the boolean[] partition of the labels for this instance (true if
          the label is present, false if absent, for each of the existing
          labels).


    - #### get

      ```
      public IdInstance get(int index)
      ```

      For test purposes only! slow!

      Parameters:
      :   `index` -

      Returns:


    - #### get

      ```
      public weka.core.Instance get(java.lang.String name)
      ```


    - #### getAllAttributeNames

      ```
      public java.util.Vector<java.lang.String> getAllAttributeNames()
      ```


    - #### getAllAttributes

      ```
      public java.util.Vector<weka.core.Attribute> getAllAttributes()
      ```


    - #### getArffFilePath

      ```
      public java.lang.String getArffFilePath()
      ```

      Returns:
      :   m\_arffFilePath


    - #### getArffId

      ```
      public int getArffId()
      ```


    - #### getAttributesArray

      ```
      public java.util.ArrayList<weka.core.Attribute> getAttributesArray()
      ```


    - #### getAttributesIndexString

      ```
      public java.lang.String getAttributesIndexString(IdInstance instance)
      ```


    - #### getAttributesNamesString

      ```
      public java.lang.String getAttributesNamesString(IdInstance instance)
      ```


    - #### getAttributesStringPool

      ```
      public uk.ac.standrews.utils.main.stringutils.StringPool getAttributesStringPool()
      ```

      Returns:
      :   m\_attributesstringPool


    - #### getByAttributesString

      ```
      public weka.core.Instances getByAttributesString(java.lang.String instanceAttributesString)
      ```

      Finds an instance having all these attributes names as present (=1)
      attributes.

      Parameters:
      :   `instanceAttributesString` -

      Returns:


    - #### getBySparseInstanceString

      ```
      public weka.core.Instances getBySparseInstanceString(java.lang.String instanceString)
      ```

      Find instances having the same sparse instance string ( indexed
      attributes string {0 1, 3 1, 5 1} including all feature and class
      attributes).

      Parameters:
      :   `instanceString` - indexed attributes string {0 1, 3 1, 5 1} including all
          feature and class attributes

      Returns:


    - #### getClassAttributes

      ```
      public java.util.Set<weka.core.Attribute> getClassAttributes()
      ```


    - #### getDbMan

      ```
      public DbManagers getDbMan()
      ```

      Parameters:
      :   `hashCode` -

      Returns:
      :   public String getByHashCode(int hashCode) { return
          this.getHash2IdMap().get(Integer.valueOf(hashCode)); }


    - #### getFirstInstance

      ```
      public weka.core.Instance getFirstInstance()
      ```


    - #### getId2IdInstanceMap

      ```
      public java.util.TreeMap<java.lang.String,IdInstance> getId2IdInstanceMap()
      ```

      Returns:
      :   m\_namesAndIdinstances


    - #### getId2WekaInstanceMap

      ```
      public java.util.TreeMap<java.lang.String,weka.core.Instance> getId2WekaInstanceMap()
      ```


    - #### getIdInstance

      ```
      public java.util.Set<IdInstance> getIdInstance(weka.core.Instance wekaInstance)
      ```


    - #### getIdInstance

      ```
      public IdInstance getIdInstance(java.lang.String identifier)
      ```


    - #### getIdInstancesByAttributesString

      ```
      public java.util.Vector<IdInstance> getIdInstancesByAttributesString(java.lang.String instanceAttributesString)
      ```


    - #### getIndexOfIdInstance

      ```
      public int getIndexOfIdInstance(java.lang.String instanceName)
      ```


    - #### getInst2LabelsPartitionMap

      ```
      public java.util.TreeMap<IdInstance,boolean[]> getInst2LabelsPartitionMap()
      ```


    - #### getInstanceNamesStringPool

      ```
      public uk.ac.standrews.utils.main.stringutils.StringPool getInstanceNamesStringPool()
      ```


    - #### getInstanceStringPool

      ```
      public uk.ac.standrews.utils.main.stringutils.StringPool getInstanceStringPool()
      ```


    - #### getInstNames2AttrIndexStringMap

      ```
      public java.util.TreeMap<java.lang.String,java.lang.String> getInstNames2AttrIndexStringMap()
      ```


    - #### getInstNames2AttrNamesStringMap

      ```
      public java.util.TreeMap<java.lang.String,java.lang.String> getInstNames2AttrNamesStringMap()
      ```


    - #### getInstSparseStringToLabelsString

      ```
      public java.util.TreeMap<java.lang.String,java.lang.String> getInstSparseStringToLabelsString()
      ```


    - #### getLabels2PartitionMap

      ```
      public java.util.TreeMap<java.lang.String,boolean[]> getLabels2PartitionMap()
      ```


    - #### getLabelsString

      ```
      public java.lang.String getLabelsString(IdInstance inst)
      ```

      Get a separated list of the labels assigned to this instance.

      Parameters:
      :   `inst` -

      Returns:


    - #### getLogFile

      ```
      public java.io.File getLogFile()
      ```


    - #### getNonZeroLabelsOfAnInstance

      ```
      public java.util.TreeSet<java.lang.String> getNonZeroLabelsOfAnInstance(weka.core.Instance i0)
      ```

      Get the names of the non-zero valued class labels of an instance

      Parameters:
      :   `i0` -
      :   `labels` -

      Returns:


    - #### getNonZeroLabelsOfTheseInstances

      ```
      public java.util.TreeSet<java.lang.String> getNonZeroLabelsOfTheseInstances()
      ```

      Get the names of the non-zero valued class labels of these instances

      Returns:
      :   the non-zero valued feature attributes of these instances


    - #### getStoredFeatureAttributes

      ```
      public java.util.Set<weka.core.Attribute> getStoredFeatureAttributes()
      ```


    - #### getToBePredicted

      ```
      public java.util.Set<IdInstance> getToBePredicted()
      ```


    - #### getTrueLabels

      ```
      public boolean[] getTrueLabels(IdInstance idInstance)
      ```


    - #### getVector

      ```
      public java.util.Vector<IdInstance> getVector()
      ```

      Returns:


    - #### getWekaInst2IdInstMap

      ```
      public uk.ac.standrews.utils.main.maputils.OneToManyMap<java.lang.String,IdInstance> getWekaInst2IdInstMap()
      ```

      Returns:


    - #### getXmlFilePath

      ```
      public java.lang.String getXmlFilePath()
      ```

      Returns:
      :   m\_xmlFilePath


    - #### labelNames

      ```
      public java.lang.String[] labelNames()
      ```


    - #### setClassAttributes

      ```
      public void setClassAttributes()
      ```


    - #### toString

      ```
      public java.lang.String toString()
      ```

      **Overrides:**
      :   `toString` in class `java.lang.Object`


    - #### getInst2PartitionMapString

      ```
      public static java.lang.String getInst2PartitionMapString(java.util.TreeMap<IdInstance,boolean[]> inst2PartitionMap)
      ```

      Parameters:
      :   `inst2PartitionMap` -

      Returns:


    - #### getNonZeroAttribIndexesOfInstance

      ```
      public static java.util.TreeSet<java.lang.Integer> getNonZeroAttribIndexesOfInstance(weka.core.Instance i0,
                                                                           java.util.Set<weka.core.Attribute> attributes)
      ```

      Only input non-class (feature) attributes if the feature attributes
      indexes are desired.

      Parameters:
      :   `i0` -
      :   `attributes` - the attributes: input only non-class (feature) attributes if
          the feature attributes indexes are desired.

      Returns:


    - #### getNonZeroAttributeNamesOfAnInstance

      ```
      public static java.util.TreeSet<java.lang.String> getNonZeroAttributeNamesOfAnInstance(weka.core.Instance i0,
                                                                             java.util.Set<weka.core.Attribute> attributes)
      ```

      Get the non-zero valued attributes of an instance

      Parameters:
      :   `i0` -
      :   `attributes` -

      Returns:


- Overview
- Package
- Class
- Use
- Tree
- Deprecated
- Index
- Help

- Prev Class
- Next Class

- Frames
- No Frames

- All Classes

- Summary:
- Nested |
- Field |
- Constr |
- Method

- Detail:
- Field |
- Constr |
- Method
